# Healthcare needs and expectations of family members caring for mental healthcare users in South Africa

**DOI:** 10.4102/curationis.v47i2.2625

**Published:** 2024-11-08

**Authors:** Takalani E. Mbedzi, Anna E. van der Wath, Miriam M. Moagi

**Affiliations:** 1 Department of Nursing Science, Faculty of Health Sciences, University of Pretoria, Pretoria, South Africa; 2 Department of Nursing, Faculty of Health Sciences, University of Limpopo, Polokwane, South Africa

**Keywords:** caring, expectations, family members, mental health care users, healthcare needs, healthcare professionals

## Abstract

**Background:**

In South Africa, many mental healthcare users reside in rural areas and 91% of them live with their family members. Exploring and describing the needs of family members caring for mental healthcare users is important to determine their expectations of the healthcare system.

**Objectives:**

The study aimed to explore and describe the healthcare needs and expectations of family members caring for mental healthcare users in Vhembe district, Limpopo province, South Africa

**Method:**

A qualitative, descriptive and contextual design was used to collect data from 16 purposively selected family members caring for mental healthcare users. Data were collected through semi-structured face-to-face interviews, and analysed using thematic data analysis to develop themes and categories.

**Results:**

The results of the interviews yielded two themes, healthcare needs and expectations of family members. The needs included physical healthcare, psychological healthcare, and social, financial, educational and spiritual needs. The healthcare expectations were divided into two sub-themes: (1) expectations from the healthcare systems, and (2) expectations from healthcare professionals.

**Conclusion:**

The study showed that support from community members and healthcare providers could reduce the burden of care perceived by family members. Results confirmed the need for developing a family psychoeducational programme adapted to the South African context to meet the family members’ needs. Quantitative research on healthcare needs with a larger sample of family members is recommended.

**Contribution:**

The study may contribute to mental health nursing practice and education. Nursing support based on the needs of family members may enhance their well-being and caregiving abilities. The importance of tailor-made psychoeducational family support should be emphasised in nursing education.

## Introduction

Since mental healthcare services were deinstitutionalised, there has been a notable change in the way that mental health treatment is provided globally. Mental healthcare users (MHCUs) were placed in the care of their families without the development of supportive community-based services, especially in low- or middle-income-countries (LMICs), where human and financial resources at the district and community levels are limited (Adu & Oudshoorn 2020:313). In many cases, family caregivers are not equipped to provide care and the deinstitutionalisation process may cause family fears, apprehension and attitudes of non-acceptance (Silva & Batista 2022:47).

In this article, the following concepts apply. According to the South African Mental Health Care Act (17 of 2002:6) a *MHCU* means a ‘person receiving care, treatment and rehabilitation services or using a health service at a health establishment aimed at enhancing the mental health status of a user’. *Family members* refer to individuals who consider themselves a family member and caregiver to a MHCU for 2 years or longer. While some *healthcare needs* require intervention, others may refer to a potential healthcare issue that should be addressed to prevent disease, illness, injury or disability (Department of Health and Social Care 2022:50).

Despite the move to deinstitutionalisation, most of the mental healthcare expenditure in South Africa is directed towards hospital-based treatment (Docrat et al. 2019:706). Inadequate funding towards community-based rehabilitative mental healthcare services leaves family members with the caregiving responsibility, which may eventually lead to readmissions (Addo et al. 2018:6).

South African families are traditionally entrusted to take care of members diagnosed with mental illness and accept caring as their obligation or moral duty (Mokwena & Ngoveni 2020:5). Caregiving is characteristic of the indigenous *ubuntu* philosophy, a homogeneous culture in southern Africa (Metz 2007:321). In African societies, families described caregiving in the context of *ubuntu,* marked by responsiveness, solidarity, concern for each other, love and interdependence (Chisale 2018:4).

In Vhembe district, the context of the study, caregiving is characterised by challenges such as stigma from the community (Mabunda et al. 2022:1). Vhembe district is located in a rural area in Limpopo province in the northern part of South Africa. Traditionally occupied by the Vhavenḓa, the Vhembe district is populated by three ethnic groups, namely, Vhavenḓa, Bapedi and Tsonga (Magwede, Van Wyk & Van Wyk 2018:57). The Vhavenda people are culturally routed and engaged in traditional cultures (Ndou-Mammbona & Mavhandu-Mudzusi 2022:2). Seeing that help-seeking behaviour is influenced by cultural beliefs about mental illness, Bila and Carbonatto (2022:543) highlighted the importance of psychoeducation for caregivers in Vhembe district.

Families contribute to the well-being of MHCUs (Dehbozorgi et al. 2023:1) and provide MHCUs with the opportunity to live a productive life in the community (Montenegro et al. 2022:18). Despite the advantages of family care, families living with a MHCU may experience a variety of stressors. Stress exacerbates the burden of care, which negatively affects physical and mental well-being (Matambela & Tshifhumulo 2021:1735). Family burden is caused by challenges arising from the caring role and includes objective and subjective burdens. Objective burden refers to practical problems such as disruptions in family functioning while subjective burden refers to family members’ psychological responses to the objective burden (Nuttall et al. 2019:68). For example, MHCUs with behavioural problems such as physical aggression and a lack of hygiene may increase the subjective burden of family members (Andrade et al. 2021:7). Caregivers older than 60 years with depression and without help from other family members, were more prone to physical, psychological and social effects (Souza et al. 2017:4).

It is essential to establish a supportive framework to reduce the burden on caregivers (Cham et al. 2022:12). The level of healthcare support a family needs depends on the burden of care and the capacity of the family to provide care (Nenobais, Jatimi & Jufriyanto 2019:47). The healthcare system must alleviate the caregiver burden through the use of supportive interventions such as psychoeducation (Okafor & Monham 2023:14), mental health education, emergency support and development of interpersonal skills such as conflict resolution (Andrade et al. 2021:8; Vukeya, Temane & Poggenpoel 2022:8). Family programmes providing healthcare education, coping strategies, problem-solving and opportunities to express experiences of caregiving were successful in improving psychological distress, family functioning and knowledge about mental illness (Haselden et al. 2019:937). Mental health education reduces the burden of care and depression in family members and enhances a therapeutic alliance with healthcare providers (Tessier et al. 2023:5).

However, the healthcare system often falls short in its supportive role towards families of MHCUs. Caregivers in Iran received inadequate knowledge about mental illness and treatment. They perceived nurses as lacking awareness about the caregiving role (Tamizi et al. 2020:5). According to South African caregivers, MHCUs were only provided with medication during follow-up visits without interventions to promote mental health and facilitate support for families (Mokwena & Ngoveni 2020:8).

### Problem statement

Successful de-institutionalisation (reintegration of MHCUs in the community) requires clear operational goals and an evaluation of the viewpoints of users, families and the workforce (Montenegro et al. 2022:17). A preliminary literature search found that most research provides information on the experiences of family members caring for MHCUs (e.g. Reupert et al. 2023; Shimange et al. 2022; Verity et al. 2021). A South African study (Ntsayagae, Myburgh & Poggenpoel 2019:8) found that family caregivers experienced frustration and required physical and emotional support because of their inability to cope with the caregiving task. Examples of recent studies conducted on the needs of family members caring for MHCUs included studies in Iran (Akbari et al. 2018), India (Kartikeyan, Majhi & Kumar 2021), Ghana (Anyoke 2019) and South Africa (Thwala, Havenga & Bester 2022), the latter referring to MHCUs with schizophrenia. Irrespective of this, few studies were conducted on the support needs and expectations of families in the African context. This knowledge gap led to the formulation of the following research question: What is needed to alleviate the care burden of family members caring for MHCUs?

### Purpose of the study

The purpose of the study was to explore and describe the healthcare needs and expectations of family members caring for MHCUs in Vhembe district, South Africa.

## Research design and methods

A qualitative, descriptive and contextual design was used as such a design is flexible, holistic, aimed at an understanding of the whole and requires qualitative researchers to become involved and use an interpretive approach (Hennink, Hutter & Bailey 2020:10; Polit & Beck 2021:463). The design enabled an in-depth understanding of family members’ healthcare needs.

### Setting

Eight community health centres in Vhembe district were selected because a high number of MHCUs make use of their services. The community health centres are staffed with multidisciplinary teams that conduct monthly outreach mental health services. The teams consist of a clinical psychologist, occupational therapist and social workers stationed at the district and regional general hospital of Vhembe district. A psychologist and medical doctor visit the facility once a week to see MHCUs referred by the primary healthcare nurses and do six-monthly reviews of medication of MHCUs on chronic medication. There is only one psychiatrist in the district who visits the hospitals every month (Mulaudzi et al. 2020:5).

### Study population and sampling strategy

The research population consisted of family caregivers of MHCUs in Vhembe district. The accessible population were the family members who attended monthly follow-up visits at the community health centres. A purposive sampling method was used to select the study participants based on their knowledge and experience of the phenomena being studied. Purposive sampling refers to selecting participants with knowledge about the research topic to ensure the richness of data (Hennink et al. 2020:93). The first author (hereafter referred to as ‘the researcher’) requested the nurses at the centres to introduce the study to potential participants, and those interested were asked to contact the researcher for an appointment.

The inclusion criteria included all family members aged 18 years and older, living and caring for a relative diagnosed with a mental condition according to the Diagnostic and Statistical Manual of Mental Disorders, Fifth Edition, Text Revision (DSM-5-TR) (American Psychiatric Association 2022) for a minimum period of 2 years (to ensure sufficient exposure to caregiving experiences), able to express themselves in English and who gave voluntary informed consent. Family members not living with the MHCU and not willing to participate in the study were excluded.

### Data collection

Data were collected from April to June 2021 by the researcher through 16 semi-structured face-to-face individual interviews (Hennink et al. 2020:41) with family members caring for MHCUs. The questions in this study were: ‘What are your healthcare needs regarding caregiving to your family member with mental illness?’ ‘What kind of support do you need to help you cope with the caregiving responsibility?’ Interview skills such as paraphrasing and reflection were used and probing questions were based on the participants’ responses, for example, regarding their expectations of support. The interviews, which lasted 30–45 min, were conducted in English at the homes of the family members in a private place at a time convenient to them. A pilot interview was carried out before actual data collection and no changes in interview techniques were required. No discrepancies between intended methods and actual data collection or ethical dilemmas were experienced during data collection.

The researcher recorded observational field notes of the non-verbal communication of participants and reflective notes on her own experiences during the interviews. A voice recorder was used with participants’ permission. Data were collected until data saturation was reached at participant number 14 with two additional participants to confirm data saturation. The interviews were transcribed verbatim by the first author.

### Data analysis

Data were analysed thematically by the first author and an independent coder following the six phases of Braun and Clarke (2023:65), namely, familiarisation with the data; generating initial codes; searching for themes; reviewing themes; defining and naming themes; and producing the report. The first step entailed listening to audio recordings and immersion in the data to get familiar with the depth and breadth of the data. Initial codes were generated by classifying phrases and words used by participants connected to the research questions. The collected data were organised to identify, refine and review two themes and eight sub-themes. The process was followed by writing up each theme and sub-theme to get the story that each theme portrayed.

The researcher kept a reflective journal throughout the study to bracket her assumptions regarding the research phenomenon and to prevent researcher bias. The researcher and the coder discussed the results and compared similarities and differences to reach consensus on the final themes and sub-themes. No major disagreements occurred.

### Trustworthiness

Trustworthiness, using the strategies of credibility, dependability, confirmability, transferability and authenticity (Polit & Beck 2021:745), was applied in the study. Credibility was ensured through prolonged engagement with family members until data saturation was reached. Member checking was not performed related to logistical challenges to meet with participants a second time. Dependability was obtained through keeping an audit trail of all research steps and decisions in collaboration with the study supervisors.

Transferability was enhanced through a detailed description of the research setting and methods to allow others to compare and evaluate the applicability of the results to other contexts. An independent coder was used to ensure that the interpretations were derived from the data and not the researchers’ biases. The researcher used reflective notes to bracket any preconceived ideas about the research phenomenon before conducting the interviews to ensure confirmability. The results were supported with participants’ quotations to obtain authenticity.

### Ethical considerations

Ethical clearance was granted by the Research Ethics Committee of the University of Pretoria (reference no.: 674/2020). Permission to conduct the study was obtained from the Department of Health, Limpopo province (reference no.: LP 2020-12-005) and Vhembe district (reference no.: SF/6). Ethical principles of informed consent, the right to privacy, confidentiality, autonomy and voluntary participation, were adhered to (Polit & Beck 2021:134). The participants benefited indirectly from the results to improve family support. Participation was voluntary to prevent coercion and adhere to justice. The informed consent forms explained the purpose and procedures of the study (such as audio recording) and the option to withdraw from the study at any time. No participants withdrew from the study. A risk foreseen was emotional discomfort and one participant needed debriefing by the interviewer and was referred for counselling. Code numbers were used to identify participants such as *P1* to maintain anonymity. Password-protected transcripts were saved according to standard practice for 15 years in the institutional repository from where they will be automatically deleted.

## Results

The results are discussed according to the demographic profile and themes and sub-themes.

### Demographic profile of participants

The study was conducted with 16 family members caring for MHCUs in Vhembe district. Eleven of the participants were females and five were males. Three were aged 20–28 years; two were 40–42 years; two were 57–59 years; five were 63–69 years, and four were 72–79 years. Regarding education, five participants had no education; three had primary, three had secondary and five had tertiary education. Four participants were single; one was unmarried; five were married; five were widows and one was a widower. One participant was employed as a nurse; one was doing an internship; one was self-employed; one was a Grade 12 scholar; six who were never employed received an old-age pension, three were retired and three were unemployed. The participants’ demographics are represented in Table 1.

**TABLE 1:** Participants’ demographic profile.

Participants	Gender	Age (years)	Marital status	Occupation	Relationship to the MHCU	No. family members	Years of care provision	Level of education
P1	F	42	Single	Nurse	Daughter	2	21	Tertiary
P2	F	40	Married	Self-employed	Wife	4	15	Secondary
P3	F	59	Married	Unemployed	Sister	5	10 >	Uneducated
P4	F	76	Widow	Old-age pensioner	Mother	3	10 >	Uneducated
P5	M	28	Single	Unemployed	Brother	2	20	Tertiary
P6	F	69	Married	Old-age pensioner	Mother	3	10	Primary
P7	F	26	Single	Internship	Sister	11	4	Tertiary
P8	M	63	Widower	Retiree	Father	3	10 >	Tertiary
P9	F	63	Single	Retiree	Sister	4	20 >	Secondary
P10	M	20	Unmarried	Scholar	Grandson	11	10 >	Secondary
P11	M	74	Married	Old-age pensioner	Father	14	10 >	Primary
P12	F	72	Married	Old-age pensioner	Mother	13	10 >	Uneducated
P13	F	79	Widow	Old-age pensioner	Mother	5	10 >	Uneducated
P14	F	65	Widow	Retiree	Mother	5	19 >	Tertiary
P15	M	57	Widow	Unemployed	Mother	3	20 >	Primary
P16	F	67	Widow	Old-age pensioner	Mother	2	20 >	Uneducated

*Source: Mbedzi*, T., 2023, ‘Family members caring for mental health care users in the Vhembe district’, PhD thesis, Department of Nursing Science, University of Pretoria

F, female; M, male; MHCU, mental healthcare user; P, participant number.

The participants were caregivers of MHCUs diagnosed with different mental conditions, including schizophrenia, bipolar disorder, substance-induced disorder and epilepsy with psychosis. The duration of the MHCUs’ conditions ranged from 4 to 20 years and 93% of the participants had more than 10 years of caregiving experience. Two family caregivers were not living in the same household but were neighbours of the MHCUs. Most of the participants were parents, seven were the mothers of the MHCUs and four were siblings.

### Presentation of the results

Two themes emerged from the results, namely, healthcare needs and expectations of family members caring for MHCUs, as presented in Table 2. Each sub-theme is substantiated with direct quotations. The number, family relationship and gender of the participant is indicated in brackets for each quotation.

**TABLE 2:** The needs of family members caring for mental healthcare users.

Themes		Sub-themes
1.	Healthcare needs of family members caring for mental healthcare users	1.1 Physical healthcare needs 1.2 Psychological needs 1.3 Social needs 1.4 Financial needs 1.5 Educational and information support needs 1.6 Spiritual support needs
2.	Healthcare expectations of family members	2.1 Expectations from the healthcare system 2.2 Expectations from healthcare professionals

*Source:* Mbedzi, T., 2023, ‘Family members caring for mental health care users in the Vhembe district’, PhD thesis, Department of Nursing Science, University of Pretoria

### Theme 1: Healthcare needs of family members caring for mental healthcare users

The healthcare needs of the family members were grouped into the following six sub-themes: (1) physical healthcare needs, (2) psychological needs, (3) social needs, (4) financial needs, (5) educational and information support needs, and (6) spiritual support needs.

#### Sub-theme 1.1: Physical healthcare needs

The physical strain of caring for a MHCU has a detrimental impact on the quality of life for the primary caregiver. Family members disclosed several health issues that they disregarded in favour of the MHCU’s medical demands. One participant, for instance, reported pain that limited her mobility:

‘Yes, I do feel pains in my body, heaviness of my body, difficulty walking, as well feeling pain when I sit down.’ (P3, sister, female)

Participant 16 sustained physical injuries when she tried to restrain the MHCU:

‘When you look at me [*removing a mask and showing the facial scars where she was bitten by the MHCU*], she bit me when I wanted to apprehend her the time, she wanted to beat me up.’ (P16, mother, female)

Another participant expressed how caring for the MHCU affected her physical health and well-being when she was diagnosed with a cerebrovascular incident, stress-related pain and exhaustion:

‘I then consulted the doctor … who told me that I am affected by stroke. I told the doctor that no, it could not be stroke, it is the heart, which is so painful. As I get worried about something, it builds up and ultimately led to a serious problem. Then I went to the clinic, they said you are suffering from stress related problems, my head was so heavy, neck pains experienced, lack of energy, so they said that is stress. They even asked me as to what is bothering me.’ (P6, mother, female)

#### Sub-theme 1.2: Psychological needs

The psychological needs relate to the demands and stress of providing long-term care to MHCUs. The participants expressed a need for psychological counselling to acquire coping strategies, and knowledge and skills to manage the MHCUs. A sister of a MHCU indicated that counselling will help her to cope with constant concerns about the MHCU:

‘Even counselling I need it because we are thinking too much. Sometimes she can decide to kill herself or kill us. So, I really want counselling. Counselling will help us to refrain from persistent memories of all these things which she is doing.’ (P12, sister, female)

Counselling services can provide guidance on how to deal with caregiving problems:

‘Now that I know you, I can be able to call and say: I am experiencing this … what must I do? She can help me by giving advice to say, do this and that, can you see?’ (P2, wife, female)

Other participants needed counselling to know more about the mental health condition of the MHCU, fostering acceptance related to the condition and to acquire coping skills such as evidenced by the following quotations:

‘I need counselling … families living with a member suffering from mental illness, if we are told how should they manage them.’ (P14, mother, female)

‘… [*W*]hen you see him being mentally ill it is very painful therefore counselling is mandatory. As you expected him to go to the university it is painful really. Counseling will help me to learn the skill of how to manage mental illness, it will assist me to learn that if this happen what must be done. Counselling will teach us as to how we can accept the situation.’ (P5, brother, male)

#### Sub-theme 1.3: Social needs

The social needs of family members were derived from negative social experiences such as social stigma, discrimination and lack of support from community members. When MHCUs relapse, the stigma and fear surrounding mental conditions stop the family members from getting help. As stated in the following two quotations, the majority of participants said that the community does not meet their needs for social support, but instead chooses to look on while they struggle:

‘That brings pressure on you since when the patient is not well, he has relapsed from his mental condition, when you look at the community members, they just watch or ask: “What is happening?” All that I have indicated brings a lot of frustration to us as a family.’ (P5, brother, male)

‘Here in the village people do not come to assist me … They do not help me anyway … they do not assist, it becomes a problem as I am alone in this struggle.’ (P16, mother, female)

One participant shared the experience of verbal abuse from street children insulting her mother and she felt victimised by people making derogatory remarks about the mental status of the MHCU. She stated that caregivers rather need respect and protection from the community:

‘Street children, they like provoking her … you can find that other families cannot reprimand their children. Instead of saying to them that this person is an adult who is suffering from mental illness who needs to be respected, they do not do that … since you do not have someone to protect you, you cannot dodge the emotional abuse, people telling you that your mother *Vha a penga* [is mentally ill, she is mad] using insulting words ….’ (P1, daughter, female)

Some participants were concerned because the MHCUs lost employment because of their mental condition, leaving them with financial difficulties. The wife of a MHCU shared experiences of her husband being stigmatised by employers and co-workers:

‘The employer and co-workers said that they could not live with him because he is mentally ill. People can say whatever they want, they talked bad things but I realized that it is because they do not know what is happening. If it can happen to them, how would they feel and how will they manage the situation?’ (P2, wife, female)

The same participant expressed a need for family members to show their support:

‘I have contacted the close relatives of my husband … I do not need anything from them; I do not need their money, just if they can come to see us to prove that they love me, to show that they are supportive.’ (P2, wife, female)

#### Sub-theme 1.4: Financial needs

The financial challenges experienced by participants reflected their financial needs to cover the costs of living such as meeting basic needs, transport costs, costs for carrying out ancestral rituals to treat mental conditions, medication expenses and burial society dues. One elderly participant used her pension funds to provide food for her two MHCUs:

‘They want to eat, when I get my old age grant money, I buy everything, *mieliemeel*, [maize flour to cook porridge], *tshisevho* [meat or vegetables to eat with porridge] with my money … these are the people I am supporting financially.’ (P4, mother, female)

Participants have to fund transport to the clinic or hospital:

‘Financial needs are another thing … Even if I can tell myself that I am going to take him to the hospital, where will I get the money to pay for transport, whose car will I get … who can offer it free of charge? No one will do that …’ (P5, brother, male)

Participants expressed a need for the disability grant to be provided permanently as they have to reapply annually, following a tedious process:

‘My prayer is that if the disability grant is permanent, it should not be cut off.’ (P13, mother, female)

Another participant shared her story of having to borrow money from unregistered money lenders to obtain traditional healthcare:

‘… I was told his illness was caused by the ancestors. I had to take him there as you know, it is the ritual to fulfil the desires of the ancestors, we were expected to buy many things … I had to go to the people who lend money so that I must do the ritual … borrowed a lump sum of money …’ (P16, mother, female)

A few participants reported that at times they had to buy medication for unemployed MHCUs from the pharmacy when there was no stock at the healthcare centres. This placed a financial burden on them as stated in the following quote:

‘You may find that there are mentally ill individuals who are not working; others have lost their jobs because of mental illness. There was a time when the department was unable to distribute the pills to the patients, we found ourselves not having money, and even the very patient himself had nothing. We had to go to the chemist shops since there was nothing we could give to the patients.’ (P5, brother, male)

Another participant reported how she felt obliged to give money to her sister to pay the community burial societies as evidenced by the following quotation:

‘When she [*MHCU*] does not have money … she will come to my place … I cannot say I do not have the money I will always assist her because my mother died who used to help her … I will take the money which was given to me and give it to her so that she can go and pay for the funeral scheme’s books.’ (P3, sister, female)

#### Sub-theme 1.5: Educational and information support needs

Most participants reported a need for mental health education as they were not given adequate information regarding the diagnosis, treatment options and management of the MHCU so that they can cope with community-based care, as evidenced in the following quotation:

‘… [*I*]f you could be told what type of illness is this? Families living with a member suffering from mental illness, how should they manage them?’ (P14, mother, female)

One participant caring for her husband wondered why the MHCU relapsed while he was taking medication and expressed a need for more information:

‘It is only that I do not understand how come if a person is taking medication like every time but still, he has this problem. It is like there is a specific time the illness should just come back? As we are aware that the medication is intended to make him stable, still you find the illness recurring … what is really happening? I am more concerned about this because I want to understand what is happening.’ (P2, wife, female)

Because of their lack of involvement in the treatment programme, participants did not know what kind of medication was provided for the MHCU. They indicated the need for information about the type of medication, the indications and the mechanism thereof. They believed this would enable them to continue to provide care:

‘… [*I*]f the nurses can come, they find me and the tell me how do some of the things. Since I never went with her to the clinic. They can let me how these pills are used and the indication, or maybe to say the pills will take so many months, thereafter they will be changed. The nurse will be health educating me so that I can realize, no matter what can happen, I must not stop supporting her.’ (P3, sister, female)

‘We know a bit about medication though I may not be able to call the pills by the names since I am not a doctor, but for example, we are told that the MHCU should drink all his pills. If one is missing, I will run to the hospital [*name of the facility removed*] to collect his pills.’ (P5, brother, male)

#### Sub-theme 1.6: Spiritual support needs

Family members expressed their spiritual needs differently based on their religious beliefs and some obtained support from traditional leaders and church leaders to help them cope with the caring burden. While it is customary in some African cultures to seek assistance from traditional healers, several participants thought that the consultations were ineffective and turned to Western medications instead. The following quotation supports this view:

‘The illness started some years back, as you know that people will always try somewhere for help before consulting the western medical doctors, but in 2002 they observed that all efforts of consulting the traditional practitioners were not working, so they then decided to consult the hospitals.’ (P5, brother, male)

The participants reported the negative attitude displayed by nurses at the hospital who tend to blame the family for consulting traditional health practitioners, rather than showing understanding, as seen in the following quotation:

‘The problem is when we tell the nurses that we visited the traditional healer they become angry and start to shout at us, blaming us saying we made the illness worse. Really nurses do not understand how it feels to have someone with mental illness in the family.’ (P8, father, male)

The participants also indicated that they were given spiritual counselling by their church pastors. They reported how the pastors supported them by providing basic means, home visits and prayers to cope better with the caring responsibility. According to the participants:

‘The pastors would pray for us when I ran short of food, they provided food, bathing and washing soap.’ (P16, mother, female)

‘Our pastors visited us and prayed for us and laid their hands on us. They visit us when the patient is not well and bring spiritual healing to the patient.’ (P12, mother, female)

### Theme 2: Healthcare expectations of family members

The healthcare expectations to meet the support needs of family members were divided into two sub-themes namely, expectations from the healthcare systems and expectations from healthcare professionals.

#### Sub-theme 2.1: Expectations from the healthcare system

Participants highlighted that the MHCUs need day care and rehabilitation services where MHCUs can participate in meaningful activities to relieve caregivers, as evidenced in the following quotation:

‘If there was a place for them where it can be said that in the morning they can wake up and bath, then leave to that place for day care. They [*MHCUs*] can be kept there for the day with gates locked accompanied by their relatives. Then in the evening they are taken home, I think it is better. I believe their illness can be manageable, as they will be busy doing something.’ (P1, daughter, female)

Care and rehabilitation services may teach the MHCUs occupational and social skills suitable to their level of functioning that may assist in the generation of income and employment opportunities:

‘It can assist with the rehabilitation of my brother’s finance where he can further his studies like doing security courses where he can get employed.’ (P5, brother, male)

The sister of a MHCU expressed that such a facility, described by her as a ‘school’, will relieve boredom and will provide an opportunity for MHCUs to interact with each other:

‘When she is doing nothing but just sitting at home it makes her to think deeply and get more worried. I think it can be better if we look for a school where she will be able to interact with other people also affected by mental problems.’ (P7, sister, female)

The inconsistent availability of psychiatric medication at community health centres contributes to families’ financial burden. Family members expect the Department of Health to ensure consistent access to adequate treatment. When medicine is unavailable, family members are responsible for the associated costs to obtain the medication, which increases the burden of care as evidenced in the following quote:

‘The Department of Health was unable to distribute the pills to the patients, we found ourselves without money, and even the patient himself had nothing … when we got to the pharmacy, it cost R400.00 and we found that the pills will only cover about a week … we have to collect the medication at [*name of district hospital*] if they are out of stock at the community health center which is expensive in terms of transport money because the hospital is far from this place.’ (P5, brother, male)

Most of the participants acknowledge that the medication should be taken to maintain the mental stability of the MHCU, but they complained about the disabling side effects, particularly the intramuscular depot treatment, which rendered the MHCUs unable to function independently. One participant indicated a need to change the MHCUs’ treatment to oral medication as the depot injections had serious side effects:

‘The injection was not good for her at that time while she was at the hospital [*name removed*] they used to give her, but she experienced some problems like drooling of saliva, tremors, and body shaking. If they can give her the injection, they will also prescribe some tablets to manage the problems of the side effects caused by the injection.’ (P7, sister, female)

‘… [*I*]f they can inject her, she will not even manage to pick up her child, because she will be paralyzed. Previously they injected her at Giyani. She came back looking like this [*demonstrating the tremors and stiffness of the muscles*].’ (P11, father, male)

#### Sub-theme 2.2: Expectations from healthcare professionals

Participants expressed a need for healthcare home visits during which healthcare professionals could evaluate how well families were managing caregiving responsibilities, the condition of the MHCU, medication adherence, side effects and substance use. They can offer the caregivers practical advice on managing problematic behaviour. The following quotes support this aspect:

‘If you can help her, I mean to say if you can come every week to assist us by checking her if indeed, she is drinking her pills as prescribed.’ (P12, mother, female)

‘Maybe the health care workers may visit the family, maybe once in three months to find out how we are coping so that we can be able to give them the report. We would get an opportunity to report indicating that we are observing this and telling them that the patient is smoking cigarettes and dagga [*cannabis*] excessively which can lessen the burden of care on our side, just like now as we are communicating with you.’ (P5, brother, male)

The participants were of the opinion that home visits will foster nurse–patient relationships, strengthening the importance of medication adherence. One of the participant reflected the following:

‘So that when she is around you can come and talk to her encouraging her to take medication. If you can advise her on the dangers of not taking her treatment.’ (P7, sister, female)

Participants believed that these visits may assist in facilitating registration for grants:

‘[*The Department of Health*] are not coming to see the MHCUs. In reality, they only see him coming to the clinic to take the treatment … When they are supposed to consider an application for the grant; they only check the list indicating how many times he takes the medication, not visiting the MHCU to see the environment where he stays. I mean when he is supposed to be given a disability grant.’ (P5, brother, male)

## Discussion

The participants were concerned about their physical healthcare needs, which are sometimes neglected. Physical problems may impair functioning and cause long-term health problems, as explained in the next studies. For example, the insomnia prevalence was high among caregivers of MHCUs, particularly in those with depression, anxiety and fatigue (Chen et al. 2023:1). Spending more time in caring negatively affected family caregivers’ well-being across all demographic groups, stressing the need to screen caregivers’ well-being regularly (Sin et al. 2021:6). In this study, 56% of participants were over the age of 60. The burden of care reduces the overall quality of life and life satisfaction, especially in older caregivers who may require special interventions (Phillips et al. 2023:18). Physiological reserves decrease in older caregivers leading to a decline in caregiving capacity; therefore they require additional instrumental support (Souza et al. 2017:9).

Sources of psychological distress mentioned by participants included a fear of being harmed by the MHCU. This is supported by other studies; caregivers who were at risk of being injured or killed by MHCUs lived in fear (Shimange et al. 2022:6), while others felt insecure while caring for MHCUs (Chen et al. 2019:5). The need for counselling services, is consistent with international studies. A literature review (Ong, Fernandez & Lim 2021:217) recommends family counselling and psychoeducation to address the family’s physical, emotional and social needs. In this study, participants anticipated that counselling would enhance mental health education and the development of effective coping skills. Pastoral counselling helps South African families to relieve stress (Modise, Mokgaola & Sehularo 2021:5), while psychological counselling empowers families to manage challenging behaviours, mobilise resources for support and learn how to manage the feelings associated with caregiving, thus strengthening coping strategies and resilience (Shimange et al. 2022:6). As recommended in a study carried out in Zimbabwe, counsellors should incorporate cultural issues such as traditional healthcare practices when planning psychological interventions (Sichimba, Janlöv & Khalaf 2022:7).

The participants’ social challenges such as social isolation, stigma and discrimination pointed to a need to address community responses towards MHCUs and their families. A cross-sectional study (Ayalew et al. 2019:10) in Ethiopia indicated that caregivers’ perceived stigma positively correlated with their burden; the longer period of contact hours to take care of the patient per day was associated with higher caregiver burden. Professionals must acknowledge family caregivers’ need for support and the importance of family relationships (Sunde, Vatne & Ytrehus 2022:1330). The family members’ need for social support aligns with the African philosophy of *ubuntu,* stating that ‘when a community is broken, the individual is also broken’ (Ewuoso & Hall 2019:99). Healing of the community cannot occur without the healing of the individual. *Ubuntu* promotes conducive relationships with others, fellowship and community friendliness (Ewuoso & Hall 2019:101). Healthcare professionals need to encourage alternative ethical frameworks such as *ubuntu to* facilitate community and psychological support and reduce stigma for family members. Promotion of reintegration and participation, and awareness campaigns in society of MHCUs were recommended to reduce stigma in a South African population (Monnapula-Mazabane & Petersen 2023:9437).

Financial challenges were reported by most research participants. As the majority of MHCUs were unemployed, their only source of financial support was their families, indicating a need to address the financial needs of MHCUs. Insufficient funds to cover transport costs to attend follow-up visits led to MHCUs defaulting treatment. This finding was supported by Ntsayagae et al. (2019:4), who showed that some MHCUs failed to follow up and caregivers sometimes sent other relatives to collect the medication. A systemic review reported that between 17% and 50% of caregivers of persons with severe mental illness in sub-Saharan Africa were unemployed (Addo et al. 2018:7). When family caregivers were asked about the type of support needed, they indicated that the social system, including financial and housing aspects, needed to be improved (Iseselo & Ambikile 2020:6). To encourage MHCUs’ social and economic involvement, it is crucial to provide them with housing, supported employment and family psychoeducation (Killaspy et al. 2022:118). Supported employment (work rehabilitation interventions) is recommended to alleviate the burden on caregivers and is beneficial to meet the vocational needs of MHCUs (Mavindidze, Nhunzvi & Van Niekerk 2023:12).

Family members reported a lack of knowledge on how to identify the signs and symptoms of relapse and the management of mental health problems. They also expressed a need for information regarding MHCUs’ diagnoses and treatment. In a South African study, only one participant out of 16 knew the psychiatric diagnosis of the patient. A lack of knowledge impairs family members’ abilities to oversee MHCUs’ treatment plans (Du Plessis et al. 2021:9). Poor knowledge and negative attitudes towards mental illness significantly increased caregivers’ perception of associated stigma (Ebrahim et al. 2020:8). Psychoeducation alleviated the burden experienced by families and should be integrated as a routine intervention for family caregivers. Content should include the aetiology, symptoms and management of mental illness coupled with problem-solving skills, communication skills and information on available community resources (Okafor & Monaham 2023:14).

Participants expressed spiritual needs based on cultural and religious beliefs and sought support from traditional and church leaders to cope with the care burden. The negative effects of caregiving can be balanced by social and religious support (Phillips et al. 2023:18). Many families use their belief system, worship and prayers to obtain internal strength through prayer (Casaleiro et al. 2022:250). In a study conducted in Uganda (Verity et al. 2021:8), a shortage of mental health resources and poor access to healthcare services caused caregivers to rely on spiritual and traditional care. Religion-based networks were perceived as social support systems; however, the participants acknowledged that some religions discouraged medication adherence (Verity et al. 2021:8). The involvement of traditional and spiritual healers can be optimised through training in mental health issues and collaboration between traditional and Western healthcare practitioners (de Diego-Cordero et al. 2023:8).

The participants stated that the MHCUs need community-based psychiatric services including rehabilitation and day care services. Azman, Singh and Sulaiman (2019:466) found that the absence of mental health services, the stigma attached to mental illness and the unavailability of rehabilitation services have a detrimental effect on the burden of family members. After attending peer support programmes, family caregivers reported improved well-being and improved healthcare systems with patient-centred care (Joo et al. 2022:903).

The participants indicated problems with the availability of suitable psychiatric medication at community health centres, despite the Department of Health (2013:29) statement that essential psychotropic drugs should be made available at all levels of care. The availability of psychotropic drugs is a serious problem affecting LMICs (Iseselo & Ambikile 2020:6–8). The availability of the required psychotropic medication is a prerequisite for the successful integration of mental health services (Wakida et al. 2019:12). In another study (Wood, Watson & Barber 2021:40–41), participants expressed frustration with hospital emergency departments and the general inadequacy of mental health system resources. They suggested the use of mobile mental health units to respond to a mental health crisis before the police are involved. Systemic barriers to access to care in this study included economic and logistical barriers, which can be addressed through policies to address healthcare disparities in underserved communities to bridge the gap in healthcare equity (Pervez & Anjum 2023:24).

The participants expressed a need for home visits by healthcare professionals to reduce the burden of care. Another study performed in South Africa rated the support caregivers received from healthcare professionals as ineffective (Raluthaga et al. 2023:n.p.). According to Zuurmond et al. (2020:50–51), home visits improved caregivers’ participation and the efficacy of psychoeducation in a study in Ghana. Quality improvement is critical for providing supportive care and calls for organisational innovations, applicable policies and the involvement of stakeholders in providing effective community-based mental healthcare (Moudatsou et al. 2021:10). An integrated care model is recommended for LMICs by Thornicroft et al. (2019:22) that comprises identification of the condition and a care package with psychoeducational information provided by a community healthcare outreach team.

### Limitations

One of the study’s limitations was the delay in data collection caused by coronavirus disease 2019 (COVID-19) limiting procedures. As a result, the participants experienced some inconvenience as the researcher had to reschedule appointments. Some participants also withdrew from the study because of the loss of close relatives and related COVID-19 bereavement processes. Another limitation was that member checking of results was not performed, which could have implicated the trustworthiness of the results. Conducting the interviews in English in a native language-speaking context was also a limitation. A strength of the study was that participants were provided the opportunity to express their needs which informed the development of a support programme.

### Recommendations

The Department of Health should formulate policies and provide the required resources for mental healthcare support. Daycare centres and agreements with accredited non-governmental organisations to provide rehabilitation services should be established. Capacitation of primary healthcare nurses is recommended to provide psychological support, mental health education, home visits and non-judgemental counselling, especially when families choose to consult traditional healers and spiritual leaders. Appropriate information should be provided to empower families in decision-making. A referral system to social workers for social grant applications and renewal processes should be in place. During home visits, nurses should assess family members for early detection of and referral for physical problems and psychological distress. Availability of the required psychiatric medication at clinics is non-negotiable.

## Conclusion

The results showed that family members caring for MHCUs have different healthcare needs and expectations from the healthcare system. The needs of the family members should be addressed by the healthcare system to reduce the family burden. Mental healthcare practitioners need to disseminate information about mental health and make appropriate referrals. Acknowledging and fostering community members’ spiritual and cultural belief systems such as *ubuntu*, may enhance caring ethics towards MHCUs and their families. The study recommends the development of rehabilitation facilities and a psychoeducation programme, tailored to address the healthcare needs of families in rural villages of Vhembe district. The recommendations will empower families to make informed decisions to overcome the challenges they encounter while providing care to MHCUs.
